# Vascularized Free Tissue Transfer in a Patient with Hemophilia B: Case Report and Literature Review

**DOI:** 10.1155/2019/5430786

**Published:** 2019-12-29

**Authors:** Mohammed Qaisi, Justin Kierce, James Murphy

**Affiliations:** ^1^Division of Oral & Maxillofacial Surgery, John H. Stroger Hospital of Cook County, Chicago, IL, USA; ^2^University of Toronto, Oral & Maxillofacial Surgery, Toronto, ON, Canada

## Abstract

Hemophilia is a blood disorder characterized by impairment of the coagulation cascade leading to an increased bleeding risk (Kauffman, 2014). As such, surgical management of these patients can become difficult and well-defined surgical guidelines are not yet in place (Assoumane et al., 2017). Close monitoring of perioperative factor levels may be even more crucial for those undergoing microvascular free tissue transfer. This is because either a hypercoagulable or hypocoagulable bleeding state has the potential to further increase the risk of vascular compromise to the flap. We report a successful case of mandibular reconstruction using a free fibular flap in a patient with severe hemophilia B and the protocols we used, as well as a review of the literature of similar cases. In the literature, we identified 6 cases of microvascular free tissue transfer in patients with hemophilia; two of these cases had complications which were both related to excess bleeding. It is crucial that these cases be managed in a multidisciplinary fashion in close consultation with a hematologist. The role of venothromboembolism (VTE) prophylaxis in the hemophilic patient undergoing free tissue transfer is discussed.

## 1. Introduction

Hemophilia is the second most common inherited bleeding disorder behind Von Willebrand's disease. There are two main forms of hemophilia: hemophilia A (1/5,000 male live births) is a deficiency of factor VIII and hemophilia B (1/25,000 male live births) is a deficiency of factor IX [1]. Ultimately, both forms of the disease are clinically indistinguishable and lead to an impairment of the coagulation cascade causing an increased tendency to bleed. The International Society of Thrombosis and Hemostasis grades the severity of hemophilia based on the concentration of circulating coagulation factors: 5-40% of normal is considered mild, 1-5% is moderate, and less than 1% is severe [2]. Patients with mild and moderate hemophilia are often asymptomatic and may go unnoticed, or not present until later in life. In comparison, patients with severe hemophilia may suffer from spontaneous major hemorrhagic events [3]. Commonly, patients with severe hemophilia experience recurrent hemoarthrosis, leading to joint destruction requiring hip and knee replacement surgeries [4, 5]. The majority of the data regarding safety of surgical procedures in hemophiliacs come from these series.

A much smaller cohort of hemophiliacs requires microvascular free tissue transfer. Microvascular free tissue transfer has a reported success rate between 95 and 99% for the reconstruction of ablative defects in the head and neck [6, 7], although the literature reporting success rates of free tissue transfer in hemophiliacs is limited to 6 case reports. Altered hemodynamics may compromise the blood flow and flap survival rate; therefore, strict protocols for microvascular surgery may be even more crucial. It is unclear whether these procedures can be performed safely in the severe hemophilic patient due to the lack of data regarding this topic. The risk of life threatening hemorrhage and flap compromise due to vascular compression from hematoma formation or from vascular thrombosis during these surgeries needs to be further investigated.

We present a case report of a patient with severe hemophilia B who successfully underwent free fibular flap reconstruction of a mandibular defect after the resection of an ameloblastoma. The aim of this report is to contribute to the body of literature regarding the safety of microvascular free flap reconstruction in hemophiliacs with the hope of generating more predictive protocols.

## 2. Report of Case

**Figure 1:**
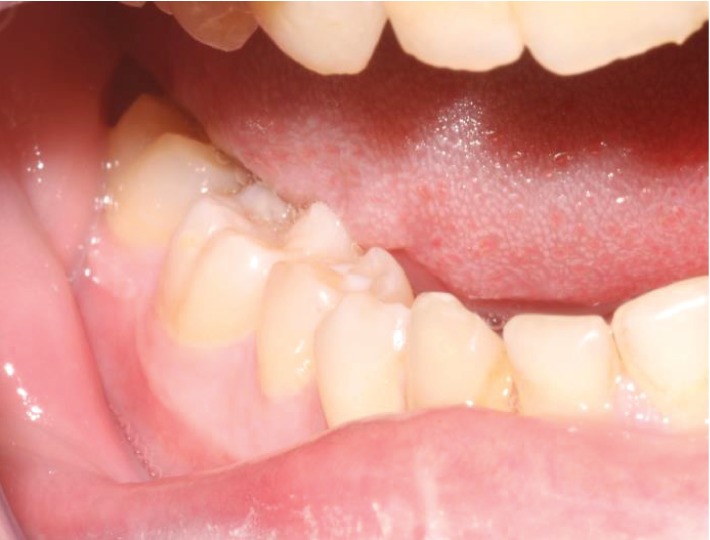
Preop intraoral photo. Buccal bony expansion is appreciated on manual palpation.

**Figure 2:**
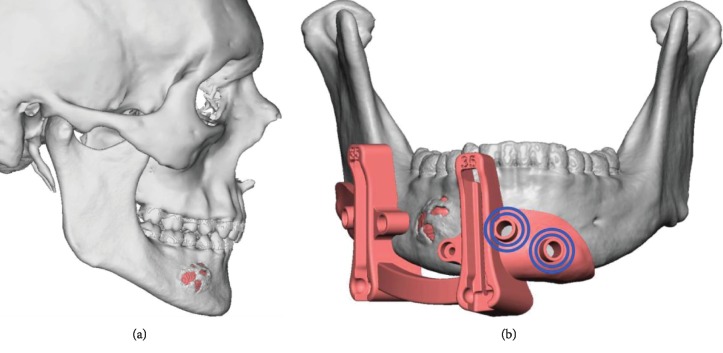
Preoperative operative 3D image of tumor involving the mandible. (a) Lateral view. (b) Frontal view: showing cutting guide and planned resection.

A 23-year-old caucasian male with severe hemophilia B presented for evaluation of an expansile mass of his right mandible (Figure 1). The computed tomography (CT) scan demonstrated a 2.5 cm by 2.5 cm hypodense, expansile lesion with cortical erosion in the right mandibular body (Figure 2). An incisional biopsy of the lesion was recommended, and the hematology service was consulted for recommendations regarding the perioperative management. The perioperative protocol set forth by hematology for the minor biopsy procedure is shown in Table 1. It involved the infusion of recombinant factor IX 30 minutes prior to the procedure followed by daily injections for a two-week period. Amicar (aminocaproic acid) was also given for a one-week period as outlined in Table 1.

The patient's preoperative factor IX assay was <1% at baseline. The patient underwent a transoral incisional biopsy of his mandibular lesion and was admitted overnight for observation. His postoperative factor IX assay was 117%, and his hospital course was uneventful. The surgical site was hemostatic postoperatively, and the patient was discharged home the following morning. The patient continued factor IX infusions at home.

**Figure 3:**
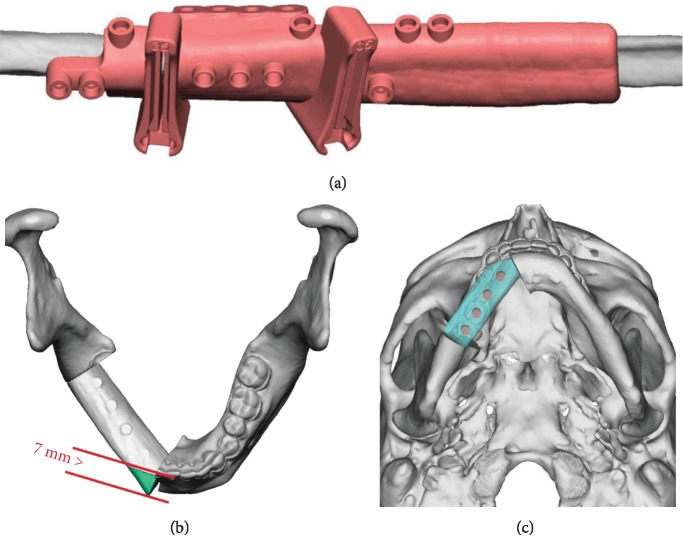
Showing virtual plan. (a) Fibula cutting guide. (b) Planned mandibular reconstruction with single segment fibula, superior view. (c) Mandibular reconstruction with fibula and immediate implants, submental view.

**Figure 4:**
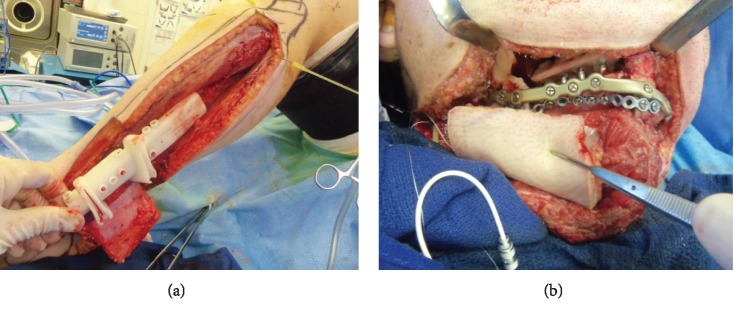
Intraoperative photos. (a) Showing fibula free flap harvest. No abnormal bleeding encountered during harvest or after release of tourniquet. (b) Fibular bone inset at the mandible via transcervical approach with good hemostasis.

The pathology from the biopsy came back as a follicular ameloblastoma. The treatment options considered included segmental resection of the lesion with 1 cm margins with either second-stage bone grafting 3-6 months afterwards, or immediate reconstruction with microvascular free tissue transfer. The hematology team recommended to treat the patient with the best reconstructive option as long as he was properly transfused with factor IX to an adequate level and was closely monitored. The patient opted for segmental resection and reconstruction with a free fibular flap with simultaneous dental implant placement and immediate prosthetic rehabilitation (Figures 3 and 4).

Given the extent of the second surgery, the recommendations for the second operation were different from those for the incisional biopsy. The recommendations are summarized in Table 2, which involved the infusion of factor IX both preoperatively and intraoperatively at more frequent intervals and with larger doses. Intraoperative redosing was necessary due to the length of surgery and factor half-life.

The patient underwent a right segmental mandibular resection through a transcervical approach, and the defect was reconstructed with a single segment fibular free flap. The patient was stable throughout the procedure with subjective mild hemorrhage. The patient was given Amicar 6 grams every 6 hours, and was infused with factor IX preoperatively with 100 units/kg, and then twice again during the remainder of the 12-hour procedure.

The patient did well postoperatively and was transfused with factor IX every 12 hours for the seven-day hospital stay. Clinical parameters (capillary refill, color, turgor, and temperature) for flap monitoring remained within normal range, and implanted flow coupler monitor (Synovis) was indicative of adequate venous blood flow. The patient was given aspirin 325 mg daily as part of our postoperative free flap protocol. The patient was also on VTE prophylaxis with 5,000 U of heparin injected subcutaneously every 8 hours. The use of VTE prophylaxis was discussed with the hematology team, and it was recommended that the patient should be treated the same as a nonhemophiliac patient as long as his factor levels were repleted to a normal range.

The patient did not experience any hemorrhagic events or flap thromboses. Drain output remained within the expected range for the procedure, and all drains were removed by postoperative day 7. Factor IX assay was measured after the initial preoperative dosage of Mononine (117%) and then on postoperative day 1 (72%). The aPTT was measured daily and ranged between 19.5 and 25.3, which was used as the primary indicator of coagulation homeostasis (relative to the preoperative aPTT).

**Figure 5:**
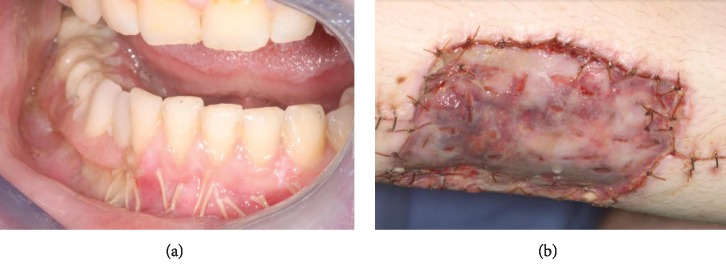
One-week postoperative photos. (a) Intraoral photo showing provisional prosthesis in place. (b) Photo showing lower extremity donor site with skin graft in place. No issues with postop bleeding.

**Figure 6:**
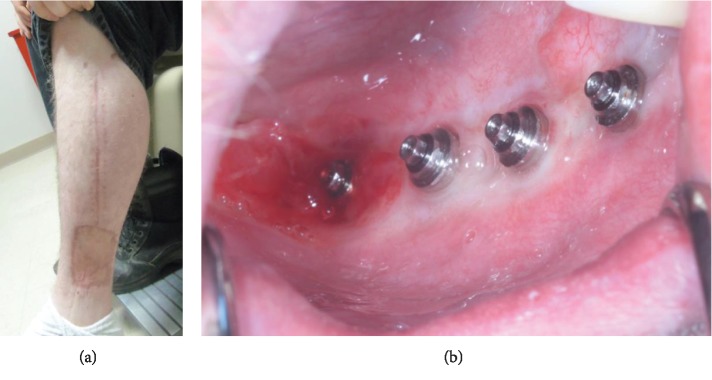
Six-month postoperative photos. (a) Lower extremity donor site. (b) Intraoral photo with prosthesis removed.

The patient was discharged home on postoperative day 7. The patient had no issues related to hemostasis on follow-up (Figures 5 and 6).

## 3. Discussion

Only a few decades ago, surgery in hemophiliacs was considered difficult and was associated with significant risks, such as uncontrolled bleeding, hemolytic complications associated with impure factor concentrates, transmission of blood-borne infections, and problems with wound healing [8, 9]. With advancements in the treatment of hemophilia and the ability to replete deficient factor levels, surgery can be performed more safely. Even with these advances in treatment, strict perioperative management is imperative [10, 11]. Due to the relatively longer surgeries and the importance of hemodynamic stability in flap survival, strict management may be even more crucial for the hemophiliac undergoing microvascular free tissue transfer. Currently, there are no specific protocols in place for these patients.

### 3.1. Factor Replacement

Preoperative assessment by a hematologist and adequate replacement of factor are important [5, 12, 13]. Goldman et al. report a series of 55 cases of major abdominal surgery in patients with hemophilia [12], where daily control of factor levels during the perioperative period was emphasized. The authors found that with adequate factor control, there was no significant differences in complication rates in the hemophilic versus nonhemophilic patients. Bastounis et al. reported a series of 68 cases of hemophiliacs undergoing general surgery procedures [13]. Of the 68 patients, there were four reported postoperative complications, two of which were related directly to bleeding. The factor replacement protocols and factor assays were not reported. Goldman et al. suggest achieving a preoperative factor of at least 50%, whereas Bastounis et al. reported maintaining postoperative factor levels above 30%. The Australian Hemophilia Centre recommends that for hemophilia A patients undergoing major surgery that factor VIII be maintained at 80-100% for the first 3 postoperative days, 60-80% for the 4-6th postoperative days, followed by a level of 40-60% for the next 7 days [13, 14]. Overall, there does not seem to be a general consensus on what is defined as adequate replenishment of factor levels during major surgical procedures. Regardless, if surgical management is performed in conjunction with a hematologist and factor levels are closely monitored, postoperative bleeding complications appear to be insignificant in hemophiliacs relative to nonhemophiliacs [5].

### 3.2. VTE Prophylaxis

Historically, it seemed counterintuitive to provide VTE prophylaxis to hemophiliacs due to their increased bleeding tendencies, though with adequate replacement of factor it can be argued that the hemophiliac can be treated the same as a nonhemophiliac. Furthermore, the potential risk of thromboembolic events has been discussed in the adequately replete patient [12, 15–19]. At a single institution, Goldman reported that 82% of hemophilic patients received VTE prophylaxis after the year 2000, but only 29% received prophylaxis between 1990 and 2000; no statistical difference was found in VTE incidence or bleeding complications between the two groups [12].

Botero et al. suggested that compression stockings alone are adequate for VTE prophylaxis in hemophiliacs. In their retrospective cohort study of 72 patients with hemophilia A or B undergoing hip or knee replacement surgery [20], all of the patients utilized compression stockings for up to 6 weeks after surgery. The authors found that the risk of VTE after hip or knee replacement surgery in patients with hemophilia A or B was 0.5–1.4%.

The use of mechanical versus pharmacological VTE prophylaxis is also variable and controversial. While Botero et al. report that pharmacological prophylaxis is not usually required for major general surgical procedures [20], VTE prophylaxis is commonplace in free tissue transfer due to the inherent risk of VTE in the setting of longer surgeries, and decreased mobilization. The difference between pharmacological and mechanical means of prophylaxis has not been explored in the microvascular patient.

Eppsteiner et al. completed a systematic review and meta-analysis in postoperative trauma patients that suggests that the benefits in terms of VTE prevention between compression and subcutaneous heparin are similar, but the risks of bleeding are far greater with heparin. The authors also conclude that the use of heparin may have benefits in certain scenarios, such as when compression is not a viable option, or in patients with microvascular anastomoses [21].

The issue of VTE prophylaxis is further complicated in the patient undergoing microvascular free tissue transfer. These patients tend to be high risk for VTE due to their concomitant risk factors, such as old age, malignant conditions, prior radiation, poor nutritional status, and long postoperative recovery periods [22]. Bahl et al. found that the incidence of VTE was significantly higher in the patient undergoing free tissue transfer. They grouped patients into low, medium, and high risk for VTE based on their Caprini risk score. In the low risk group in particular, the authors found that the risk of VTE was 4.6% in the free tissue transfer patient versus 0.3% in all other patients [22]. In general, the risk-to-benefit ratio is difficult to objectively assess for each patient. The hemophiliac undergoing microvascular free tissue transfer only adds a third layer of complexity to the issue. Currently, there are no objective measures to determine the need for VTE prophylaxis and use of prophylaxis needs to be determined on an individual patient basis based on subjective measures.

### 3.3. Anticoagulation in Free Tissue Transfer

Due to the possible risk of thrombosis at the pedicle anastomosis, some surgeons advocate the use of various anticoagulation regimes, though this is largely based on anecdotal data and experiences [23]. Chien et al. reported that 96% of reconstructive surgeons utilize some form of anticoagulation regimen to manage free tissue transfer patients [24]. Various anticoagulants that are administered include aspirin, heparin, low molecular weight heparin, and dextran [23]. The patient in this report received daily aspirin 325 mg. We suggest that if hemophilic patients have their factor deficiencies adequately replenished, they can be treated the same as a nonhemophilic patient with regard to antiplatelet therapy.

### 3.4. Review of the Literature

While it is not a free tissue transfer, Lee and Shim report the successful reconstruction of a heel defect with microvascular pedicled reverse sural artery flap in a patient with mild hemophilia B. The authors emphasized the importance of adequate factor replacement and to be prepared for the potential risks of hemorrhage, hematoma formation, and infection, as well as the potential risk of hypercoagulation [17].

In the literature, we identified 6 cases documenting free tissue transfer in patients with hemophilia A or B (Table 3). Ostric et al. report a case in which a patient required latissimus dorsi free flaps (LDMF) for the reconstruction of the right and left knee during two separate occasions. During one of the surgeries, a hematoma developed at both the donor and graft site that each required surgical evacuation. The authors concluded that the timing of the recombinant factor VIII bolus was more important than initially believed, and it is important to replace the factor before, during, and after surgery [25].

Özkan et al. report a case of an anterolateral thigh (ALT) flap for the reconstruction of an ankle defect in which a hematoma developed 1 week after surgery in conjunction with the halting of factor replacement. The authors stress the importance of close monitoring and factor replacement for 10-14 days after surgery to prevent such complications [19].

Only 2 of the 6 cases in the literature had complications, and they were both related to hematoma formation from excessive bleeding and apparent insufficient replenishment of factor.

Only the study by Knott et al. reported the use of an anticoagulant in which they used 325 mg of aspirin daily [16].

To our knowledge, our case report is the only case report of free tissue transfer to the head and neck in a patient with any form of hemophilia. In conjunction with the hematology service, a factor replacement protocol was customized for perioperative management which included replenishment with recombinant factor IX and the utilization of aminocaproic acid. The patient was thoroughly monitored for the duration of the protocol through postoperative day 21. We also utilized subcutaneous heparin to prevent VTE due to the length of surgery and extended period of immobilization. We did not experience any complications of hemorrhage or bleeding during either of the surgeries.

In conclusion, we report a successful microvascular free tissue transfer for the reconstruction of an ablative head and neck defect. We emphasize that a multidisciplinary approach in conjunction with a hematologist is necessary to develop a patient-specific protocol for adequate surgical management. This includes strict preoperative, perioperative, and postoperative management. Hemophilic patients with adequate factor replacement may receive VTE prophylaxis similar to nonhemophilic patients in cases of free tissue transfer. This case and others published show that hemophilia patients can be safely treated. We hope that this study can be used to further develop protocols for the management of hemophilic patients undergoing free tissue transfer.

## Figures and Tables

**Table 1:** The recommended perioperative protocol established by the hematology department at our institution for an incisional biopsy of a mandibular bony lesion.

(i) Infuse 100 units/kg (100%) of Mononine 15-30 minutes prior to the procedure through peripheral vein access
(ii) Check factor IX level and aPTT 15-30 minutes post-infusion (do not wait for level to come back to begin surgery)
(iii) Admit the patient for 24-hour observation
(iv) Infuse 50 units/kg (50%) of Mononine IV daily
(v) In the morning, obtain factor IX trough level (prior to infusion) and maintain trough level of 50-60%
(vi) If stable, discharge the patient with peripheral IV in place so that he can administer factor at home
Home schedule:
(vii) Infuse 50 units/kg (50%) of Mononine daily for 1 week, every other day for the following 2 weeks, then as needed for bleeding
(viii) Amicar 3,000 mg PO q6h for 7 days with the first dose the night before procedure
(ix) Follow up with the bleeding disorder clinic one week after biopsy

**Table 2:** The recommended perioperative protocol established by the hematology department for ablation of a mandibular tumor with free fibular flap reconstruction.

(i) Infuse 100 units/kg (100%) Mononine 15-30 minutes prior to the procedure through peripheral vein access. Start Amicar 6 g every 6 hours for 5 days
(ii) Obtain factor IX assay and aPTT 30 minutes postinitial infusion (do not wait for level to come back to begin surgery). This should be drawn with a peripheral stick. Do not draw through a heparinized IV
(iii) Infuse 100 units/kg (100%) Mononine 6 hours after initial dose
(iv) For subjectively increased bleeding during surgery and a prolonged aPTT, infuse 100/units/kg Mononine as needed
(v) Infuse 25 units/kg (25%) Mononine 12 hours after the 2nd dose and then 25 units/kg every 12 hours while admitted
(vi) Obtain factor IX assay trough prior to 3rd dose then as needed while admitted to maintain trough level > 50% through to the 14th postoperative day. This should be drawn with a peripheral stick. Do not draw through a heparinized IV. If factor IX level < 50%, give 100 units/kg Mononine and then continue 25 units/kg every 12 hours
(vii) Discharge the patient with peripheral IV in place so that he can administer factor at home
Home schedule:
(viii) Infuse 100 units/kg (100%) Mononine once every 24 hours until postoperative day 14, and then infuse 25 units/kg Mononine every 24 hours until postoperative 21
(ix) Follow up with the bleeding disorders clinic one week after discharge

**Table 3:** A summary of the case reports in which free tissue transfer was carried out in a hemophilic patient. The table summarizes the type and severity of hemophilia, as well as the type of flap. It also reports whether VTE prophylaxis or anticoagulation was used and any complications encountered. LDMF: lattisimus dorsi musculocutaneous flap; ALT: anterolateral thigh flap; FFF: free fibular flap.

Author	Hemophilia type	Hemophilia severity	Flap #	Flap type	VTE prophylaxis/anticoagulation	Complications
Ostric et al. [25]	A	Unknown	1	LDMF	Unknown	Hematoma
Ostric et al. [25]	A	Unknown	1	LDMF	Unknown	None
Özkan et al. [19]	A	Severe (1%)	1	ALT	None	Bleeding and hematoma
Özkan et al. [19]	A	Moderate (5%)	1	ALT	None	None
Caroll et al. [26]	A	Unknown	1	LDMF	None	None
Knott et al. [16]	B	Mild (28%)	1	LDMF	ASA 325 mg qd	None
Qaisi et al. (2019)	B	Severe (1%)	1	FFF	Heparin 500 U SC TID, ASA 325 mg qd	None
